# Optical trapping with nanostructured optical fibers and motility analysis of *Pseudomonas aeruginosa*

**DOI:** 10.1007/s00249-025-01775-7

**Published:** 2025-07-08

**Authors:** Eric Faudry, Jochen Fick

**Affiliations:** 1 Université Grenoble Alpes, CNRS, CEA, Institut de Biologie Structurale, UMR 5075, Team Bacterial Pathogenesis and Cellular Responses, 71 Avenue des Martyrs, 38000 Grenoble, France; 2 Université Grenoble Alpes, CNRS, Institut Neél, 25 Avenue des Martyrs, 38000 Grenoble, France

**Keywords:** Optical tweezers, *P. aeruginosa*, Bacteria motility, Fresnel lens fibers

## Abstract

The study of bacteria swimming behavior or their interaction with other bacteria or cells requires an efficient and flexible tool for bacteria manipulation. Optical tweezers have been shown to be perfectly adapted for this task. Here we report optical trapping of pathogen *Pseudomonas aeruginosa* bacteria using optical fiber tweezers with dedicated nanostructured optical fibers. Well-aligned straight chains of up to ten bacteria were observed with optical fiber tips, whereas contactless trapping was realized at distances of 100 and 45 µm for Fresnel lens fibers and TIROFs, respectively. Very efficient trapping at laser powers as low as 3.7 mW was achieved. The bacteria vitality is an important parameter in trapping experiments. Mean square displacement and speed autocorrelation methods were applied to obtain a vitality measure and to classify the free bacteria trajectories into free floating, running, and run-wrap-run categories. The high frame rates of our observation videos allow us to reveal a relation between bacteria speed and bacteria orientation oscillations.

## Introduction

Since its development by Ashkin et al. (1986), optical tweezers have found many applications in different research domains such as biology, chemistry, or physics. In biology, optical tweezers have been used, for example, for the manipulation of living cells or biomolecules to elucidate their activity (Kishimoto et al. 2022). Optical trapping was applied for the study of bacteria motility, body, and flagella frequencies (Li et al. 2022; Min et al. 2009; Armstrong et al. 2020), or for biofilm manipulation (Camba et al. 2024). To obtain a more complete description of the swimming behavior of bacteria, including direct observation of flagella motion, the flagellum can be made fluorescent (Turner et al. 2016). The investigated bacteria include *Escherichia coli*, *Photobacterium phophoreum* (Li et al. 2022), or *Bacillus subtilis* (Bhat et al. 2022). However, to our knowledge, no work specifically dedicated to optical trapping of *Pseudomonas aeruginosa* has been published.

*Pseudomonas aeruginosa* is a pathogenic bacterium (Gellatly and Hancock 2013). Its volume-swimming behavior is controlled by a single polar flagellum (Wu et al. 2021). A review on the motility of polarly flagellated bacteria can be found in Thormann et al. (2022). The swimming behavior of *P. aeruginosa* is quite different near the surfaces, with curved trajectories near a monolayer of cells of the substrate where the Type IV pili influences swimming (Golovkine et al. 2016b). Moreover, tracking of *P. aeruginosa* simultaneously in volume and at a solid–liquid interface has revealed that the two flagella stators are crucial for the adaptation of motility in proximity of a solid surface (Hook et al. 2019)

In any *P. aeruginosa* population, motile or running bacteria coexist always with floating bacteria that do not actively swim. The bacteria are commonly switching between these two states. Furthermore, floating bacteria have to be distinguished from dead bacteria. Different microscopic methods have been developed to measure the mobility of trapped bacteria (Li et al. 2022) and were applied, for example, to measure the effect of antibodies on the bacteria speed (Apolinario et al. 2024). The bacteria trajectories can also be studied in 2D and 3D by holographic microscopy (Vater et al. 2014). It was found that 2D observation is generally sufficient to determine the motility of bacteria (Acres and Nadeau 2021).

*Pseudomonas aeruginosa* shows a characteristic run–reverse or run–reverse–pause swimming pattern. Recently, by simultaneously tracking the position of the cell body and the conformation of its flagellum, this mode was more precisely defined as a “run – wrap – reverse” mode, where the flagella is wrapped around the cell body during the intermediate state (Tian et al. 2022).

Most optical trapping experiments are run using beam-focusing tweezers integrated into an optical microscope (Li et al. 2022). In parallel, complementary approaches such as integrated photonic crystal cavities (Villa et al. 2024) or optical fiber-based tweezers Li et al. (2021) were also developed and applied for bacteria manipulation and characterization. Optical fiber tweezers have the advantage that bacteria are automatically aligned in the observation plane, which can, for example, be advantageous in propulsion force measurements (Armstrong et al. 2020). Moreover, beam-focusing tweezers require the use of high NA immersion microscope objectives. Their short working distance can be a problem in trapping far from any interface. Finally, the development of a great panel of micro-structured optical fibers allows to adapt the trapping beam properties to the trapped object (Gonzalez-Hernandez et al. 2023)

In the present paper, we report optical trapping of living *P. aeruginosa* bacteria with our optical tweezers setup, which was initially developed for the optical characterization of trapped single fluorescent nanoparticles (Decombe et al. 2013; Kumar et al. 2022). Optical fiber tips and optical fibers with two types of 3D printed diffractive optical elements were applied for highly efficient optical trapping (Asadollahbaik et al. 2022). A straightforward method to determine the bacteria motility, based on the speed autocorrelation, was developed and allowed us to check if optical trapping alters the bacteria vitality.

## Experimental methods

### *Pseudomonas aeruginosa*

The *P. aeruginosa* CHA and CHA $\Delta fliC$ strains were grown overnight in Lysogeny Broth (LB) medium at 37 $^\circ$C with 300 rpm shaking. Overnight cultures were then diluted to DO$_{600}$ = 0.05 in fresh LB medium and incubated in the same condition for 3 h to reach the exponential growth phase. The strain CHA $\Delta fliC$ lacks the flagellin FliC, and thus does not harbor a flagellum (Golovkine et al. 2016a).

### Fiber optical tweezers setup

**Fig. 1:**
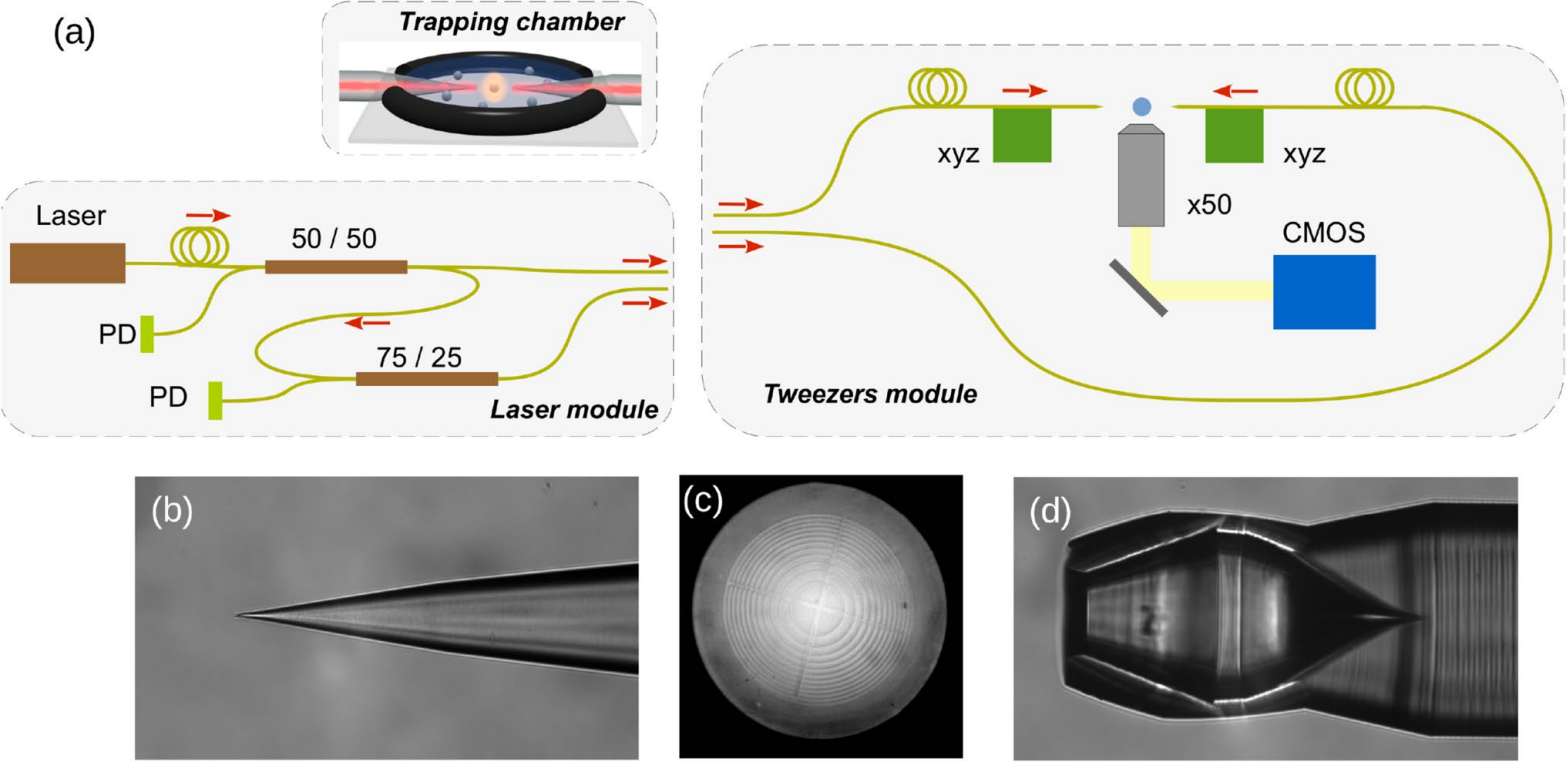
**a** Schematic of the all-fibered tweezers setup specifying the trapping chamber, the laser module, and the tweezers module which is installed inside a biological safety cabinet (PD: photodiode). **b–d** Optical microscope images of the three fiber types used for trapping: **b** optical fiber tip, **c** front view of Fresnel lens fiber, and **d** TIROF.

Our optical tweezers setup allows independent trapping with each of the two optical fibers as well as trapping in a dual-beam configuration by aligning the two fibers (Fig. 1a). A mayor constraint working with *P. aeruginosa* is the imperative to perform experiments in a biological safety cabinet (BSC) and in a class P2 laboratory. To fit inside the BSC, the optical tweezers setup was split into two modules: a very compact tweezers module fitting on a $750 \times 450$ mm^2^ breadboard and a light source module. These two modules are only connected by two optical fibers. The light source module includes a pigtailed diode-laser ($\lambda = 808$ nm, $P_{max}=250$ mW) which is coupled to two successive $2\times 2$ fused fiber couplers. The first 50:50 coupler distributes the light energy into the two fiber branches, whereas the second 75:25 coupler allows measurement of the light power transmitted between the two fibers.

Inside the tweezers module, the two trapping fibers are fixed on two sets of *xyz* inertial piezoelectric positioning systems. The trapping chamber consists of a rubber o-ring, cut into two parts and sealed between two glass slides by vacuum grease. Its dimension of $h =1.7$ mm and $d = 4$ mm ensures optical trapping far from any perturbing surface. Trapping sequences are observed through a 50x long working distance microscope objective coupled to a CMOS camera. Typical videos are recorded at frame rates of 200–1000 fps and contains typically 3000–5000 frames.

Three distinct micro-structured optical fiber types with specific emission properties are used (Fig. 1b–d). Fiber tips are obtained by chemical wet etching of commercial single-mode fibers (Nufern S630-HP) (Decombe et al. 2013). The emitted beam is of Gaussian shape with a divergent emission angle of $8^\circ$ (NA = 0.07) in water.

Fresnel lens fibers were specifically designed to obtain a converging beam for enhanced trapping efficiency (Asadollahbaik et al. 2020). The fibers used in this work produce a tightly focused Gaussian spot with a waist of 0.8 µm at a focal distance of $f=100$ µm in water and a numerical aperture of NA = 0.5. They consist of a Fresnel lens which is printed by femtosecond two photon lithography on commercial single-mode fibers (Nufern 780-HP) .

Finally, Total Internal Reflection Optical Fibers (TIROF) were developed for single fiber optical trapping. Their emission consists of an annular beam focused at $d= 45$ µm from its end and a numerical aperture of NA = 1.0. The TIROF are also elaborated by femtosecond two photon lithography on 780-HP fibers.

The high quality of the Fresnel lens fiber and the TIROF allows us to run all trapping experiments with the same two respective fibers. No degradation of the trapping efficiency or bacteria deposition was observed even after a great number of trapping experiments.

## Numerical methods

### Optical trapping

To study the trapping properties of our fiber optics tweezers, the trapping efficiency $\kappa$ is calculated. In the first step, the bacteria trajectories are obtained from the experimental videos. For this purpose, these videos are first cropped to a region surrounding the trapped bacteria using the Fiji platform (Schindelin et al. 2012). For each frame, the bacteria image is then fitted to a two-dimensional ellipse of Gaussian shape. As a result, we obtain time series of the bacteria position in axial (*x*) and transverse (*y*) directions relative to the optical fiber axis, the bacteria short and long axis and, in case of elongated bacteria, its orientation. In some cases, the bacteria image consists of two bright spots in close contact. For these “twin-bacteria”, a dedicated fitting function of two circular Gaussian spots at distance *d* is applied. The numerical tracking tools, such as the further tools presented in this work, are based on specifically developed python scripts.

Optical tweezers are described in good approximation as an overdamped harmonic oscillator. The oscillator stiffness $\kappa$ (also called spring constant) is widely used to characterize the trapping efficiency. Applying Boltzmann statistics in the framework of the equipartition theorem, the position probability distribution $P(\zeta )$ of a trapped bacteria is described by the Gaussian function:

1 $$\begin{aligned} P(\zeta ) = Ne^{\frac{-\kappa \cdot \zeta ^2}{2k_BT}}, \end{aligned}$$

with $\zeta = \{x,y\}$, *N* a normalization factor, and $k_BT$ the thermal energy (Decombe et al. 2013). The axial (*x*) and transverse (*y*) positions distributions are determined separately, thus allowing us to calculate the corresponding efficiencies independently.

### Bacteria trajectories

A complementary tracking tool was developed to simultaneously track all the bacteria visible on a video. For each video frame, the bacteria positions are roughly determined using the blob_doh function of the scikit-image python package (van der Walt et al. 2014). The focal depth of our microscope is about 1 micron. Only bacteria close to the focal plane are represented as bright spots with clear contours. Out of plane bacteria, imaged as dark spots, will not be considered.

**Fig. 2:**
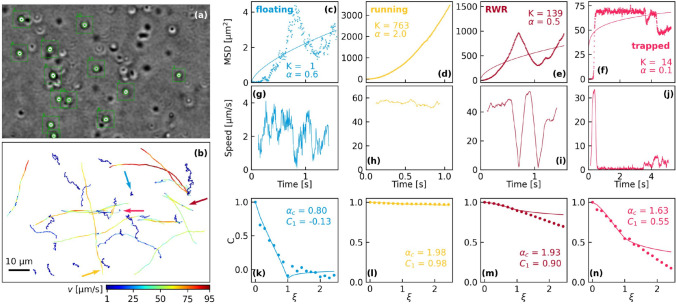
**a** Full-frame video image. The green spots and squares indicate the bacteria positions and the applied fitting areas, respectively. **b** Bacteria trajectories of the video. The four bold arrows indicate the four selected bacteria trajectory types presented in (**c–n**). The colors correspond to the names given to the trajectory types (floating, running, RWR, and trapped). **c–n** First row: mean square displacement, second row: instant speed, and last row: mean bacteria speed autocorrelation as a function of the normalized time $\xi$

In a second step, the exact position, spot size, and orientation are obtained for each selected bacterium by fitting to a two-dimensional ellipse of Gaussian shape similar to the methods used for single bacteria (“Optical trapping” section). This fit is limited to a cropped region around the bacteria position to save computational time (green rectangles in Fig. 2a). In the last step, the bacteria trajectories are constructed by searching for neighbor bacteria positions in successive frames applying a limit of three times the spot size. Trajectories expanding over at least 200 frames are selected for further treatment. The bacteria speed is calculated by multiplying the trajectory position steps by the video frame rate (Fig. 2b).

The bacteria trajectories can be classified into four main types (Fig. 2): “floating” for low speed trajectories without distinct propagation direction, “running” for higher bacteria speed with clear direction, “Run – Wrap – Reverse” (RWR) for trajectories including one or more sudden, characteristic direction changes (Tian et al. 2022), and “trapped” for bacteria which are optically trapped during at least a part of the trajectory. The floating trajectories can be attributed to non-motile bacteria, dead bacteria, or bacteria in a temporary stop mode. Moreover, RWR trajectories are a special case of the running ones. A last type named “escaped” consists in the free motion after an optical trapping sequence. It is worth to mention that, in one video, a single bacterium can show different types of trajectories.

The bacteria trajectories are investigated in the framework of fractional Brownian motion (FBM) (Metzler et al. 2014). FBM is a stochastic process with a stationary Gaussian probability distribution function (PDF) of (anti)correlated steps where the memory kernel can be tuned via a single parameter, the so-called Hurst coefficient $0<H<1$. For $H<1/2$, successive steps are anti-correlated leading to an anti-persistent sub-diffusive motion, corresponding to particle motion in a visco-elastic media. $H=1/2$ represents the familiar Brownian motion with no memory. For $H>1/2$, successive steps are correlated, leading to persistent, super-diffusive motion.

For each bacteria trajectory, we have a set of N two-dimensional bacteria position vectors $[\textbf{r}_{1}, \textbf{r}_{2}, \textbf{r}_{3}, \ldots \textbf{r}_{N}]$ with $\textbf{r}_{i} = [x_i,y_i]$ and $i = 1 \ldots N$ the respective video frame number. The time lag between two positions *i* and *j* is defined as $\tau = k\Delta t$ with $k = j-i$ and $\Delta t = 1/$fps the time interval between two successive video frames.

The mean square displacement (MSD) for a specific lag time $\tau = k\Delta t$ is defined as (Rehfeld and Weiss 2023):

2 $$\begin{aligned} MSD(\tau )=\left( \textbf{r}_{1+k}-\textbf{r}_1\right) ^2. \end{aligned}$$

The MSD frequently shows a power law described by

3 $$\begin{aligned} MSD(\tau ) = 2dK\tau ^\alpha , \end{aligned}$$

where *d* denotes the spatial dimension ($d=2$ in the present case), $\alpha$ is the scaling exponent, and *K* is the generalized diffusion coefficient. For $\alpha = 1$, Eq. (3) describes the standard Brownian motion with $D=K$ the diffusion constant. In the generalized case, *K* has the somehow strange units of an area per fractional time [µm/s$^\alpha$], which, in the following, will be omitted. The value of $\alpha$ yields a first information of the stochastic process underlying the bacteria trajectory: while $\alpha = 1$ indicates normal Brownian diffusion, the cases $\alpha < 1$ and $1<\alpha <2$ correspond to sub- and super-diffusion, respectively.

The MSD of four representative trajectories as indicated by a colored arrow in Fig. 2b are shown in (c)–(f) together with the mean bacteria speed (g)–(j) averaged over the time interval $\delta t = n\Delta t$:

4 $$\begin{aligned} \textbf{v}_j^{(n)} = \dfrac{\textbf{r}_{j+n}-\textbf{r}_j}{\delta t}, \end{aligned}$$

with $n=25$. For the floating bacteria, the speed is quite low and the MSD is not steadily increasing, resulting in a low diffusion coefficient ($K=1$) and scaling exponent ($\alpha =0.6$). The high speed of the running bacteria ($v\approx 55$) leads to nearly three orders higher diffusion coefficients of $K = 763$ and a scaling exponent of $\alpha = 2$. For the running bacteria with sudden direction changes (RWR) or for partially trapped bacteria, fitting to power law of Eq. (3) is not very meaningful. The high bacteria speed results, however, to a significantly enhanced diffusion coefficient of $K=139$ with respect to the floating bacteria in (c).

In a further step, we look at the bacteria speed autocorrelation, which can be defined as (Rehfeld and Weiss 2023):

5 $$\begin{aligned} C(\tau ) = \dfrac{\sum _{j=1}^{N-n-k}\textbf{v}_{j+k}\textbf{v}_{j}}{\sum _{j=1}^{N-n-k}\textbf{v}_{j}^2}. \end{aligned}$$

Here $\tau = k \Delta t$ is the lag time for the autocorrelation calculation. Since the interval $\delta t$, used for calculating the mean speed (Eq. (4)), can be freely chosen, it is convenient to rescale the lag time to a dimensionless time $\xi =\tau /\delta t = k/n$. With this definition, correlations due to the sliding average all appear for $\xi <1$. The speed autocorrelation function can be analytically predicted for FBM processes as (Metzler et al. 2014):

6 $$\begin{aligned} C^{FBM}(\xi ) = \left\{ (\xi +1)^{\alpha _c} + |\xi -1|^{\alpha _c} - 2\xi ^{\alpha _c}\right\} /2. \end{aligned}$$

The subscript *c* was added to the scaling coefficient $\alpha _c$ to distinction from $\alpha$ as introduced in Eq. (3). For practical purpose, the time scale $\delta t$ needs to be chosen in such a way that a time-averaging over the trajectory is not spoiled by insufficient statistics (small *n*) while still allowing for a fine sampling of $\xi$ (great *n*). In the following, *n* will be chosen to $n=7$ which seems a good compromise and results in eight data points in the $\xi = [0,1]$ interval. The value $C(\xi =1)\equiv C_1$ is supposed to be negative for sub-diffusive and positive for super-diffusive FBM processes, respectively.

The speed autocorrelation functions of the four already presented trajectories are shown in Fig. 2k–n. The negligible memory of the floating bacteria results in a negative autocorrelation value $C_1 = -0.1$ and a small exponent of $\alpha _c = 0.8$. For the running bacteria, the autocorrelation is very high with $C_1 = 0.98$ and $\alpha _c = 1.98$. The calculated speed autocorrelation is averaged over the entire trajectory. The two direction changes in the RWR trajectory (m) result in a lower autocorrelation with respect to the running trajectory. It remains, however, significantly higher than for the floating one. Finally, fitting the partially trapped trajectory turns out to be without great interest (Fig. 2n).

Continuous wavelet technique (CWT) was applied to obtain the time-dependent frequency of the bacteria orientation oscillations. The main idea of this technique is to define a so-called complex Morlet wavelet as the product of a complex sine wave and a Gaussian window (Bhat et al. 2022; Cohen 2019)

7 $$\begin{aligned} w = e^{i2\pi f t}\cdot e^{-4ln2\cdot t^2/h^2}, \end{aligned}$$

with *h* being the FWHM of the Gaussian envelope. *h* can be estimated for a required number of cycles $n_c$ by $h = n_c\sqrt{2ln2}/(\pi f)$. As a function of the specific trajectory features and length, typical values of $h = 0.1 - 0.2$ will be chosen. This wavelet is then convoluted with the time signal. The result is a complex signal. Its absolute values indicate the presence of the wavelet frequency with a time resolution determined by the wavelength width. Time–frequency information is obtained by scanning the actual wavelet frequency. Their is a clear trade-off between high frequency resolution with long wavelets and high temporal resolution with short wavelets. This point is particularly important for low frequencies of slowly running bacteria, or in case of rapid frequency changes related to RWR direction changes. In our case, it is difficult to get reliable results for frequencies below 10 Hz or better time resolutions than 50 ms.

## Optical trapping results

**Fig. 3:**
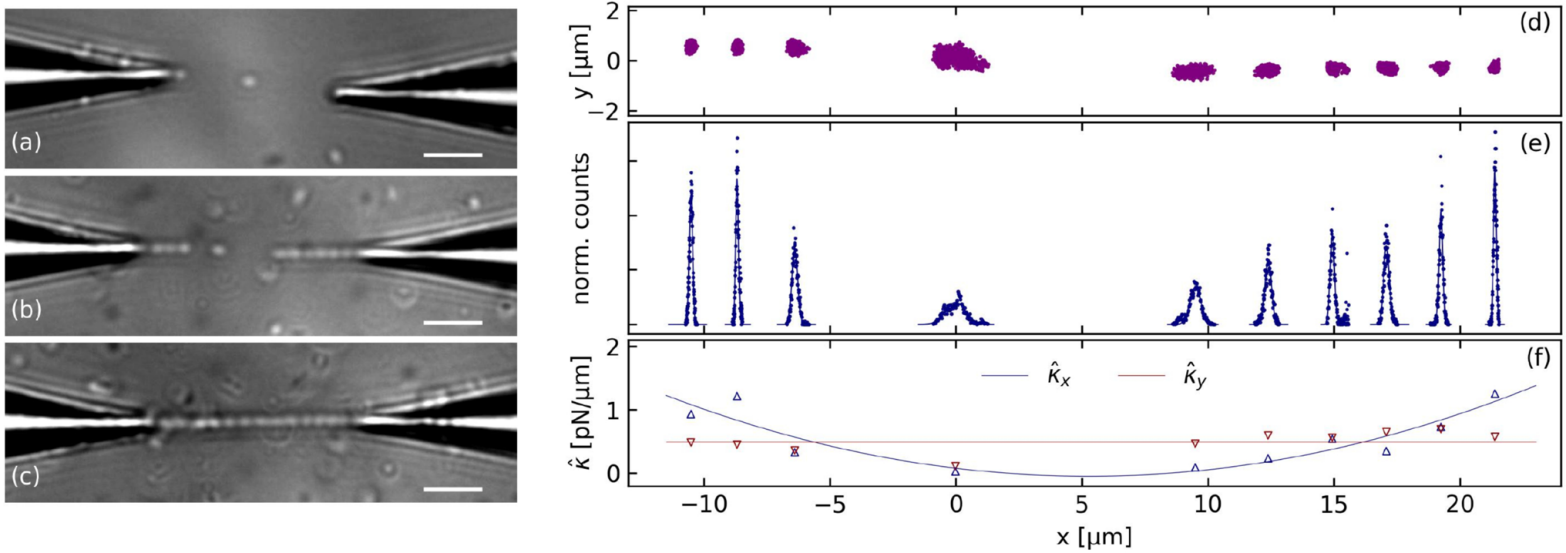
Optical trapping of *P. aeruginosa* using fiber tips. **a–c** Successive trapping sequences resulting in a bacteria chain waveguide between the two fiber tips. **d–f** Numerical treatment of trapping sequence shown in (**b**): **d** $x-y$ position clouds of the trapped and aligned bacteria, **e** normalized axial position distribution, and **f** calculated axial and transverse trap stiffness

Single bacteria could be trapped in between two fiber tips (Fig. 3a). For a fiber to fiber distance of $d\approx 25$ µm and an optical power of $P=21$ mW, trap stiffnesses of $\kappa _x = 0.01$ pN/µm and $\kappa _y = 0.03$ pN/µm are measured in axial and transverse directions, respectively.

The optical trapping is stable. However, in a time lapse of some minutes, supplementary bacteria are attracted by each of the trapping fibers, resulting in well-aligned bacteria chains. After some minutes, the two chains are joining to build a chain in between the two fibers (Fig. 3b, c).

The trapping sequence depicted in Fig. 3b with one isolated trapped bacterium and two bacteria chains of, respectively, three and six bacteria was analyzed in more detail (Fig. 3d–f). The position clouds and the axial position distributions of all ten bacteria are shown in (d) and (e). Due to the mechanical contact, the bacteria which are part of the two chains are significantly less moving than the isolated bacterium. Moreover, the bacteria close to the fiber tips are less moving than those at the chain loose ends. To quantify this feature, the effective trap stiffness $\hat{\kappa }$ was calculated using Eq. (1), being aware that the harmonic approximation is only valid for the bacterium outside the chains. The axial trap stiffness $\hat{\kappa }_x(x)$ can be roughly approximated by a parabola corresponding to exponential decrease in the light intensity, whereas the transverse stiffness $\hat{\kappa }_y(x)$ is quite constant with exception of the trapped bacterium.

Trapping of chains containing up to 10 bacteria was obtained without any optimization for optical powers of 63 mW. The chains are quickly disappearing after switching off the laser. Up to four bacteria remain trapped in a chain when moving the fiber in transverse direction at a speed of up to 10 µm/s.

Stable trapping of one to three bacteria is observed with one single Fresnel lens fiber for light powers in the 8–44 mW range. Similar behavior is observed with the TIRO fiber. In this case, the bacteria are trapped at 45 µm from the fiber and for light powers as low as 3.7 mW.

**Fig. 4:**
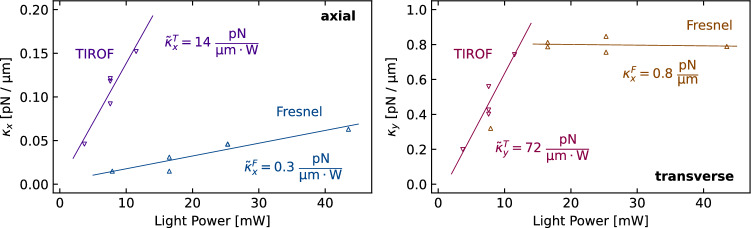
Measured optical trapping efficiencies in axial and transverse directions using TIROF and Fresnel lense fibers

For both fiber types, a series of trapping sequences of the same bacterium at different light powers was performed (Fig. 4). The normalized trapping efficiency $\tilde{\kappa }$ was calculated by linear regression. In the case of the Fresnel fiber, the axial normalized trapping efficiency is $\tilde{\kappa }_x^{F} = 0.31$ pN/µm/W. In transverse direction, the trapping efficiency is found to be constant ($\kappa _y^{F} = 0.81$ pN/µm) for light powers above $\approx 15$ mW. In the case of the TIRO fiber, the behavior is linear in both directions with values of $\tilde{\kappa }_x^{T} = 13.6$ pN/µm/W and $\tilde{\kappa }_y^{T} = 71.9$ pN/µm/W, respectively.

The possibility of manipulating trapped bacteria was verified in a further step. In case of Fresnel fibers, bacteria remain trapped for transverse translation speeds of up to $v_y=40$ µm/s. Moreover, bacteria transfer from one Fresnel fiber to a second Fresnel fiber or to a TIRO fiber was realized without any difficulty.

## Bacteria motion analysis

**Fig. 5:**
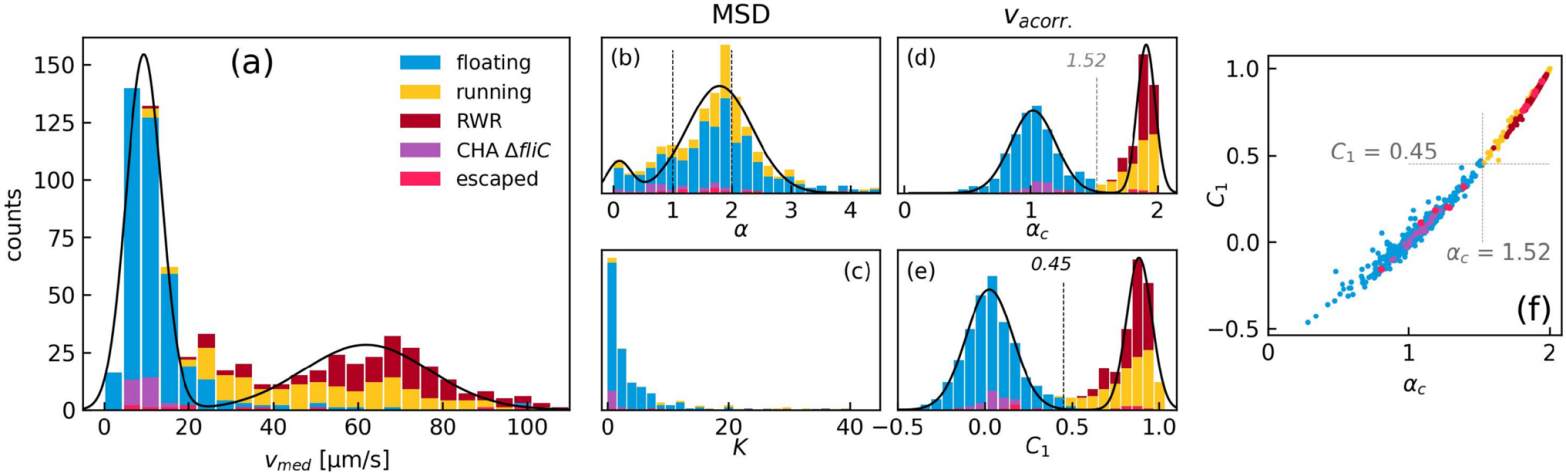
Statistics of 724 bacteria trajectories. **a–e** Distribution histograms of the mean bacteria speeds, $\alpha$ and *K* from the MSD calculation, and $\alpha _c$ and $C_1$ from the speed autocorrelation. Histograms of cumulated data from floating, running, RWR, CHA $\Delta fliC$, and escaped bacteria are represented by different colors. The lines correspond to double-Gaussian fits of the respective global population distributions. **f** 2D plot of $C_1$, $\alpha _c$. Each data point represents one trajectory

One important question is if optical trapping is influencing the vitality and, more particularly, the motility of the trapped bacteria. To establish robust criteria about their motility, the maximum speed, the mean square displacement, and the speed autocorrelation were calculated for 631 bacteria trajectories recorded in 42 different videos (Fig. 5). Roughly half of the trajectories are of floating type (357). The remaining ones are evenly divided into running (148) and RWR (143) trajectories. Bacteria of the CHA $\Delta fliC$ strain, instead of the CHA strain, are used for control and comprise 26 further trajectories. As this strain is modified to have no flagella, their trajectories can be attributed to the floating class. Finally, 12 trajectories of escaped bacteria are added to investigate the influence of optical trapping on the bacteria motion. The total number of 724 trajectories allows us to make a reliable statistical treatment.

The ratio of observed floating/running trajectories does not reflect the ratio of slow/fast bacteria inside the sample solution. Slower bacteria remain for a longer time in the observation volume ($266\times 266\times 20$ µm$^3$) than fast bacteria. As we apply a mimimum trajectory length of 200 frames, a great number of running bacteria will, thus, be eliminated.

In the first step, the floating and running bacteria trajectories can be distinguished by their speed. The median speed histogram reveals two maxima, one well defined at 9.4 µm/s and a broad one around 62 µm/s (Fig. 5a). The histogram reveals that the slow speed peak contains mainly floating trajectories including CHA $\Delta fliC$, whereas the broad peak corresponds to running trajectories including the RWR ones. However, a significant overlap between fast floating and slow running bacteria trajectories is observed in the 20–40 µm/s range.

In the second step, the MSD is calculated and fitted to Eq. (3) to extract the scaling coefficient $\alpha$ and the generalized diffusion constant *K* (Fig. 5c, d). As the RWR trajectories cannot be described by this equation, they are omitted in Fig. 5b, c. The scaling coefficient allows us to identify damped Brownian motion for $\alpha \le 1$, which, in the present case, corresponds to 17% of all trajectories. $\alpha = 1$ describes a trajectory with constant speed, whereas $1<\alpha < 2$ (36%) and $\alpha >2$ (47%) are trajectories with decreasing and increasing speed, respectively. We would like to point out that $\alpha$ is only dependent on the speed variations and not on the actual speed value. The distribution of $\alpha$ shows a clear peak at $\alpha = 1.8$, indicating slightly more trajectories with decreasing than with increasing speed. The generalized diffusion coefficient *K* shows a certain distinction between slow and fast trajectories. 92% of the fast and 93% of the slow trajectories are, respectively, above and below K = 10. Moreover, all CHA $\Delta fliC$ trajectories are below 10.

In a third step, the speed autocorrelation gives the most appropriate tool for separating running and RWR from floating trajectories. Both the speed autocorrelation value $C_1$ and the exponent $\alpha _c$ show thresholds of $C_1=0.45$ and $\alpha _c=1.52$, respectively (Fig. 5d–f). Trajectories above these values can be attributed to motile bacteria. As the autocorrelation is averaged over the entire trajectory, even multiple direction changes do not modify the final result. Furthermore, all CHA $\Delta fliC$ trajectories are below these thresholds. Finally, the escaped trajectories are nearly equally distributed on both sides of the threshold values.

**Fig. 6:**
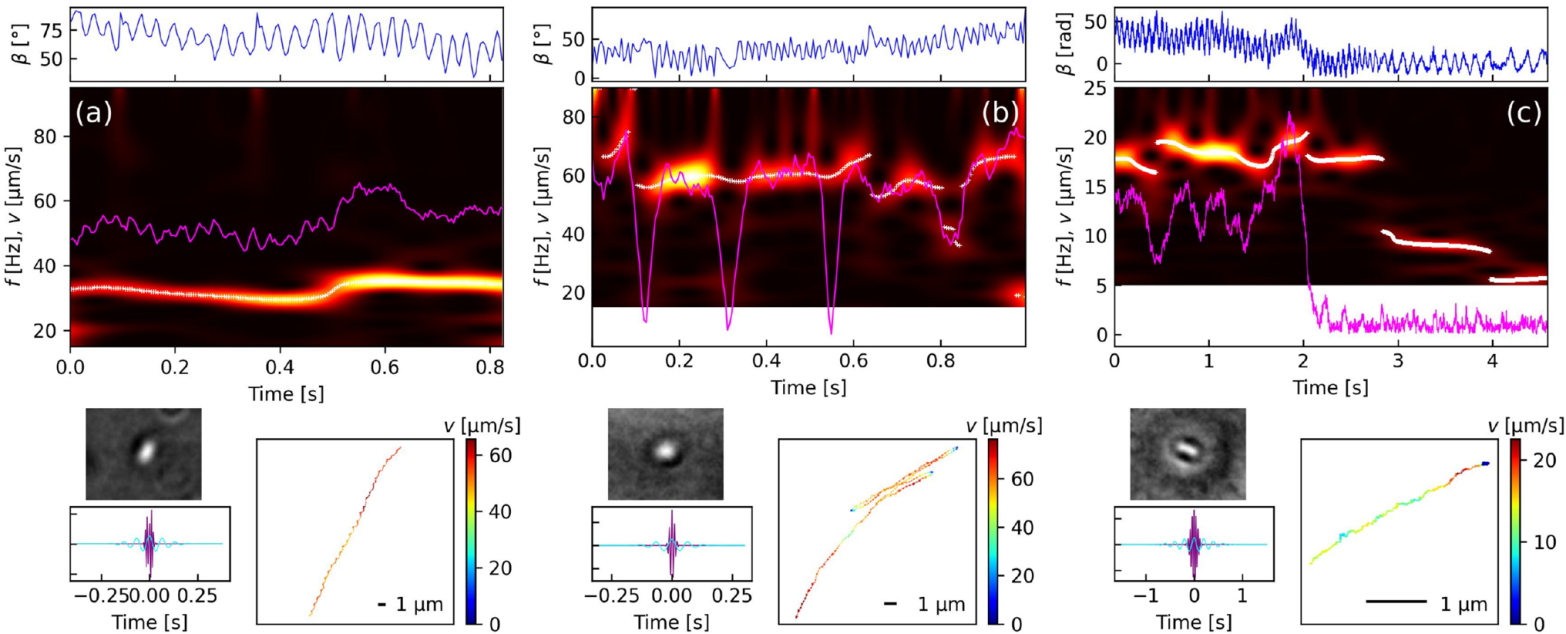
Time–frequency study of three different trajectory types: **a** running bacteria, **b** running bacteria with three RWR direction changes, and **c** bacteria getting trapped. From the top: bacteria orientation (angle $\beta$), time–frequency color map, video frame of the actual bacterium, applied wavelet at lowest (cyan) and highest (magenta) frequency, and bacteria trajectory. The white crosse and the magenta line in the color map represent the peak frequency and the bacteria speed, respectively

Periodic fluctuations of the bacteria position, size, and orientation were observed for a majority of the running and RWR trajectories. The most significant signals are obtained from the bacteria orientation, which, in fact, is not biased by possible vibrations caused by the BSC. As an example, the case of a bacterium crossing the video from the bottom to the top is depicted in Fig. 6a. The bacteria orientation is oscillating at frequencies of 30–40 Hz resulting in a wobbling trajectory. The time-dependent evolution of its oscillation frequency is very similar to the evolution of the bacteria speed, whith a small but clear increase at *t* = 0.5 s.

In case of sudden direction changes, the limited resolution of the CWT method does not allow to resolve the abrupt speed variations (Fig. 6b). The direction change is, however, clearly visible by a gap in the frequency signal. The limited time response of the speed curve in this plot is due to an averaging over 50 ms, Which is necessary to limit the noise.

Figure 6c shows the trajectory of a twin-bacterium which got trapped before finishing the separation into two daughter cells. The larger size results in slower speed and frequency. When getting trapped at $t\approx 2$ s, its speed drops to zero and the bacterium gets aligned parallel to the optical axes ($\beta =0^\circ$). Interestingly it is still oscillating when getting trapped. In the first second at about 18 Hz before decreasing to 9 Hz and a subsequent decrease to 5.5 Hz after one further second.

## Discussion

Stable and reproducible optical trapping of *P. aeruginosa* bacteria was obtained with three different nanostructured fiber types. Lowest trapping efficiency was observed for optical fiber tips in a dual-fiber configuration. In this case, the bacteria were attracted towards the tips, building bacteria chains of up to 10 bacteria. This feature, named optical binding, is well studied (Bowman and Padgett 2013; Forbes et al. 2019) and was already observed for other species such as *L. acidophilus* bacteria (Xin et al. 2013). In short, each bacterium is acting as a converging lens, creating a further trap position. The bacteria chains can be considered as an effective optical waveguide. As a consequence, a sudden increase in the optical power transmitted between the two fiber tips is observed when a last bacterium is bridging the gap between two separate bacteria chains (Fig. 3c).

Stable trapping with only one Fresnel fiber was observed for low optical power down to 8 mW. In former work, two Fresnel lens fibers in dual-fiber geometry were necessary to trap, i.e., luminescent nanorods (Kumar et al. 2022) or living algae (Asadollahbaik et al. 2022). However, trapping with only one optical fiber is of paramount interest as it makes any optical alignment superfluous. Moreover, it allows easy manipulation of trapped bacteria and independent trapping of two bacteria using the two fibers of our tweezers setup. In fact, single fiber trapping is possible as the focal spot size of the NA = 0.5 Fresnel lens is very well adapted to the oblong bacteria shape of about $1 \times 2$ µm^2^.

The high optical trapping efficiency of the Fresnel lens fiber results in a plateau of the transverse trapping efficiency at $\kappa _x = 0.8$ pN/µm (Fig. 4b). This unexpected feature is caused by our experimental sensitivity limit. The vibrational noise which is mostly caused by the BSC was estimated by measuring the position fluctuation of a fiber tip. The measured distribution width of $\sigma _x^{tip}=47$ nm and $\sigma _y^{tip}=63$ nm are of the same order than the bacteria position distribution of $\sigma =71$ nm corresponding to $\kappa = 0.8$ pN/µm. We can, thus, estimate that we are not able to measure trapping efficiencies above this value.

The TIROF was specially designed for single fiber trapping. Its annular beam emission together with its high numerical aperture (NA = 1) results in very efficient bacteria trapping ($\tilde{\kappa }_y=72$ pN/µm/W) at low light intensities of down to 3.7 mW. In axial direction, its trapping efficiency is more than 40 times higher compared to the Fresnel lens fiber.

To get trapped, a free bacterium first passes near the light beam between the optical fiber and the trapping position. Then it is pushed into the trapping positions at measured speeds of 80–150 µm/s corresponding to optical forces of 0.7–2 pN. The great focal length of the Fresnel lens fiber ($f=100$ µm) allows us to capture free bacteria in a large volume. For the TIROF with $f\approx 45$ µm, the capturing efficiency is lower. The most effective trapping feature, thus, consists in first trapping a bacterium by a Fresnel fiber before transferring it to a TIROF.

The optical trapping force $F_{opt}$ has to be compared with the force $F_{flag}$ the bacteria flagella is producing to swim at speed *v*. For low Reynolds numbers, this force can be calculated by $F_{flag}=\gamma _0v$, with $\gamma _0$ the Stokes drag coefficient. Describing the bacteria as a rigid prolate spheroid with minor and major semi-axis of $a\approx 0.5$ µm and $b\approx 1$ µm, the drag coefficient can be estimated by $\gamma _0 = 4\pi \eta \cdot b/\{ln(2b/a)-1/2\}\approx 1.42\cdot 10^{-8}$ Ns/m, with $\eta$ the dynamic viscosity of water (Chattopadhyay et al. 2006). As an example, running at 20 µm/s requires a propulsion force of $F_{flag}=0.3$ pN. This value is of the same order than propulsion forces measured on *E. coli* bacteria (Armstrong et al. 2020).

We wonder if the flagellum propulsion force is sufficient to allow a trapped bacterium to escape. In our experiments, escaped bacteria are observed to swim at speeds of up to 30–100 µm/s, i.e., their flagella generate forces of 0.4–1.4 pN. In case of stable trapping, this force has to be compensated by the optical restoring force. The axial restoring force for optical trapping with a TIROF at 10 mW laser power can be calculated by $F_{opt} = \Delta x \cdot 0.14$ pN, where $\Delta x$ is the bacteria position shift from the optical trap center. Thus, a propulsion force of 0.4 pN would cause a position shift of 2.9 µm. In real experiments, such a large shift would, however, result in bacteria escape. Therefore, one can suppose that trapped bacteria are either not motile, in a temporary pause state, or having their flagella motion inhibited by the optical trap.

For a running bacterium, the flagella rotation leads to bacteria body rotations which in turn results in periodic fluctuations of the bacteria position and orientation (Tian et al. 2022). In a great number of trajectories, the orientation fluctuation could be clearly identified as harmonic oscillations and are well visible in the time–frequency plot (Fig. 6). In some cases, a wobbling trajectory can even be observed (a). The body frequency varies in general with the bacteria speed and RWR events are visible as a gap in the frequency plot (Fig. 6). In general, there is a clear relation between the bacterium orientation frequency and its speed. The body rotation is slower than the flagella rotation. In the case of *E. coli*, body frequencies of 10 Hz were measured for flagella frequencies of 100 Hz (Min et al. 2009). Our measured frequencies are much higher than the body rotational frequencies below 1 Hz measured by another group for *P. aeruginosa* running at comparable speeds (Tian et al. 2022). This difference is probably related to our higher video recording rate of approximately 200 fps.

A bacteria population contains always a part of dead specimen which are floating due to the Brownian motion. In optical trapping experiments, they are more easily attracted than motile bacteria running at speeds of up to 100 µm/s. To some extent, the motility of a bacterium is related to its vitality. To check the motility, three specific parameters calculated from the bacteria trajectories have been presented. They are the generalized diffusion coefficient *K* from the MSD, and the speed autocorrelation exponent $a_c$ and value $C_1$. An important feature is that these parameters are not significantly biased by the sudden RWR direction changes, which are characteristic of highly motile *P. aeruginosa*. A classification using the MSD exponent $\alpha$ was, however, found to be impossible.

To investigate the influence of optical trapping on the bacteria vitality, we investigated the trajectories of 12 un-trapped bacteria (Fig. 5). After the trapping sequence, half of them were running with speeds comparable to those of free bacteria. Three of them showed even at least one RWR event. The remaining six bacteria showed a floating trajectory. As the bacteria state before trapping is not known, we cannot conclude on the influence of optical trapping on the bacteria state. We can, however, conclude that at least for the six escaped bacteria with a running trajectory, optical trapping of some tens of seconds did not alter their vitality. Moreover, as the proportion of floating/running trajectories after trapping is roughly the same as observed for all investigated trajectories, we suggest, that, in general, the optical trapping has no significant influence on the bacteria vitality .

## Conclusion

Stable and reproducible optical trapping of living *P. aeruginosa* bacteria has been achieved using our optical fiber tweezers in a single fiber configuration. Trapping was contactless at distances of 100 and 45 µm from original Fresnel lens fibers and TIROFs, respectively. The use of these nanostructured fibers permits very efficient trapping and manipulation at laser powers as low as 3.7 mW. The study of free bacteria trajectories allowed us to set up robust parameters to evaluate the bacteria motility based on the speed autocorrelation. Bacteria released or escaped from the optical trap did not show degradation of their motility.

Trapping of living bacteria with a single fiber is of great interest to design further experiments to study single bacteria interaction with eukaryotic cells. Few works reported the use of optical trapping to depict the interactions of large and non-motile yeast fungi and bacteria with eukaryotic cells (Guimarães et al. 2019; Tam et al. 2010; Kemper et al. 2013). Because of the small size and high motility of many pathogenic bacteria, carrying out this type of experiment could be highly challenging. In this context, our optical trapping approach based on the use of lensed optical fibers holds a significant contribution to overcoming this challenge.

## Data Availability

Data and python scripts will be made available on request.
